# Multimodal Light Microscopy Approaches to Reveal Structural and Functional Properties of Promyelocytic Leukemia Nuclear Bodies

**DOI:** 10.3389/fonc.2018.00125

**Published:** 2018-05-25

**Authors:** Christian Hoischen, Shamci Monajembashi, Klaus Weisshart, Peter Hemmerich

**Affiliations:** ^1^Leibniz Institute on Aging Research, Jena, Germany; ^2^Carl Zeiss Microscopy GmbH, Jena, Germany

**Keywords:** live cell imaging, fluorescence fluctuation microscopy, super-resolution, promyelocytic leukemia, tumor suppressor, oncogene

## Abstract

The *promyelocytic leukemia* (*pml*) gene product PML is a tumor suppressor localized mainly in the nucleus of mammalian cells. In the cell nucleus, PML seeds the formation of macromolecular multiprotein complexes, known as PML nuclear bodies (PML NBs). While PML NBs have been implicated in many cellular functions including cell cycle regulation, survival and apoptosis their role as signaling hubs along major genome maintenance pathways emerged more clearly. However, despite extensive research over the past decades, the precise biochemical function of PML in these pathways is still elusive. It remains a big challenge to unify all the different previously suggested cellular functions of PML NBs into one mechanistic model. With the advent of genetically encoded fluorescent proteins it became possible to trace protein function in living specimens. In parallel, a variety of fluorescence fluctuation microscopy (FFM) approaches have been developed which allow precise determination of the biophysical and interaction properties of cellular factors at the single molecule level in living cells. In this report, we summarize the current knowledge on PML nuclear bodies and describe several fluorescence imaging, manipulation, FFM, and super-resolution techniques suitable to analyze PML body assembly and function. These include fluorescence redistribution after photobleaching, fluorescence resonance energy transfer, fluorescence correlation spectroscopy, raster image correlation spectroscopy, ultraviolet laser microbeam-induced DNA damage, erythrocyte-mediated force application, and super-resolution microscopy approaches. Since most if not all of the microscopic equipment to perform these techniques may be available in an institutional or nearby facility, we hope to encourage more researches to exploit sophisticated imaging tools for their research in cancer biology.

## Introduction

The *promyelocytic leukemia* (*pml*) gene is a target of the t(15;17) chromosomal translocation, which fuses *pml* reciprocally with retinoic acid receptor α (RARα) (1). PML protein is the major building unit of the so-called PML nuclear bodies (PML NBs). PML NBs appear as nuclear dot-shaped structures that are interspersed between chromatin (2). PML NBs are heterogeneous and dynamic structures, ranging in size from 0.1 to 1.0 µm, and typically there are 5–30 bodies per nucleus, depending on the cell type, phase of cell cycle, and the cellular stress level (3–5). Overexpression of PML in cancer cell lines induces cell cycle arrest and apoptosis (6). *PML* knock-out mice develop a range of cancers including papillomas, carcinomas, and lymphomas after exposure to carcinogens (7). Furthermore, loss of PML is a hallmark of human cancers from diverse tissues (8). Therefore, PML is regarded as a potent tumor suppressor in *in vitro* (biochemistry, cell culture experiments) and *in vivo* (model organisms). At the physiological level, PML has been functionally linked to anti-inflammatory and antiviral response pathways, metabolism, stem cell maintenance, and aging, while more mechanistically, PML’s role in tumor suppression is linked to control of the cell cycle, apoptosis/senescence, cell migration, angiogensis, and the DNA damage response (9, 10). Since upon DNA damage PML NBs accumulate various DNA damage response factors and physically associate with damaged chromatin, they have also been suggested to play important roles in genome maintenance, probably by supporting specific aspects of DNA repair pathways (11–18).

A better understanding of the biophysical and biochemical mechanisms by which PML and/or the PML nuclear bodies participate in genome maintenance is expected to facilitate the development of therapeutic strategies for the treatment of PML-related diseases (19).

Novel microscopy methods have become key tools for studying biological systems over the past decades. Deep insight with unprecedented spatial and time resolution has been obtained for many cellular factors as a result of the rapid development of optical microscopy, fluorescent probes, and new labeling techniques (20). Since most biochemical mechanisms on the cellular level are dynamic by nature and cannot be fully understood by simply measuring fixed structures it is desirable to investigate the molecule of interest in real time, in living cells, at single-molecule, nanometer, and nanosecond resolution. Is this feasible? We think the answer is yes and the purpose of this report is explaining why.

We set out here to summarize our view of PML nuclear body function and assembly, the current status of powerful imaging methods and describe in some detail how the new imaging tools work in deciphering structural and functional aspects of PML nuclear bodies. Many of these tools may be accessible at a near-by imaging facility of most laboratories. We therefore wish to encourage those researchers in the fields of cancer biology to exploit the new methods more rigorously. Ultimately, the combination of classical biochemical approaches with dynamic methods and live cell imaging platforms may make it possible to fully elucidate the biophysical mechanisms underlying the structure, function, and networks of tumor suppressors and oncogenes, thus aiding the development of new therapeutic approaches.

## PML and PML Nuclear Bodies

**Figure 1:**
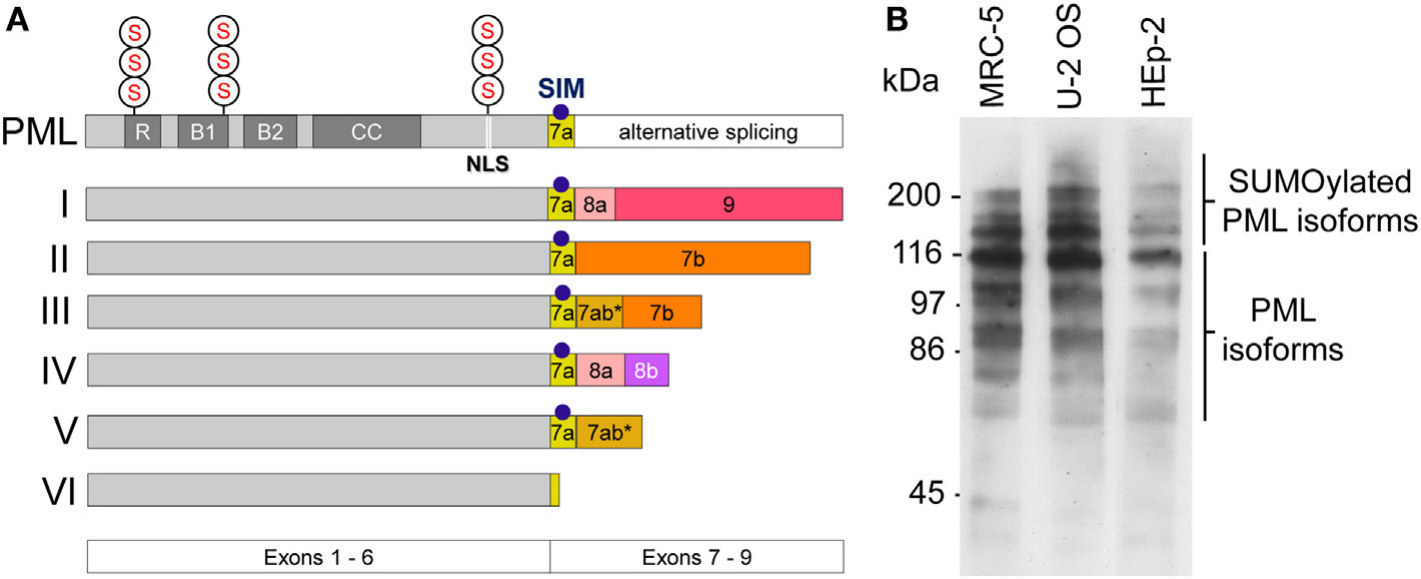
Promyelocytic leukemia (PML) protein isoforms. **(A)** Schematic depiction of the six nuclear PML isoforms (I to VI). Exons 1–6 are shared by all isoforms while their C-termini are individually different due to alternative splicing of exons 7–9. R, RING domain, B, B box; CC, coiled coil domain; NLS, nuclear localization sequence; SIM, SUMO-interacting motif; S, SUMOylation sites at arginine positions K65, K160, and K490. **(B)** PML protein expression in various cell lines. Western blot of whole cell lysates derived from MRC-5 (primary human lung fibroblasts), U2OS (Osteosarcoma-derived ALT cell line), and HEp-2 (human epithelial non-ALT cancer cell line) using a rabbit-anti-PML antibody (ABD-030, Jena Bioscience, Germany) at 1:500 dilution. Non-SUMOylated PML isoforms are detectable between 55 kDa and 110 kDa. Poly-SUMOylated PML isoforms are detected above 110–250 kDa.

The *pml* gene product (PML) is a member of the tripartite motif (TRIM)-containing protein superfamily. In human cells, six nuclear PML isoforms (I–VI) are expressed. The various isoforms originate from alternative mRNA splicing of exons 7–9 while exons 1–6 are shared by all isoforms (Figure 1A) (21). This primary sequence complexity of PML protein expression allows for common as well as individual functional modalities among the isoforms (22). PML I is the longest isoform (882 amino acids), while PML VI (552 amino acids) is the shortest isoform in the cell nucleus. Similar to other members of the TRIM family, all nuclear PML protein isoforms contain a conserved TRIM/RBCC motif consisting of a RING domain, two B-box domains and a coiled-coil domain (RBCC) (Figure 1A) (23). A nuclear localization signal (NLS) mediates predominant nuclear localization of PML. All PML isoforms contain three well-characterized small ubiquitin-related modifier (SUMO) modification sites at position 65, 160, and 490 of the PML primary sequence (Figure 1A) (24). Generally, SUMO modification of proteins plays important roles in diverse cellular processes, including chromatin organization, transcription, DNA repair, macromolecular assembly, protein homeostasis, trafficking, and signal transduction (25). In the case of PML, SUMO-2 and SUMO-3 can form heteropolymeric poly-SUMO chains (26). PML isoforms as well as their poly-SUMOylated variants can be easily detected by Western blotting (Figure 1B) (27). Additional posttranslational modifications (PTMs) of PML include phosphorylation, acetylation, and ubiquitination, all of which may serve to fine-tune PML (nuclear body) function through multiple mechanisms (28). A common feature of TRIM/RBCC proteins is homo-multimerization which generates a variety of subcellular structures including ribbon-like structures, cytoplasmic or nucleopasmic filaments, as well as cytoplasmic or nucleoplasmic bodies (23). Indeed, all six nuclear PML isoforms, when ectopically overexpressed, individually form nuclear bodies even in the absence of endogenous PML (29), with some isoforms contributing not only to nuclear body morphology (27, 30) but also function (31–33).

**Figure 2:**
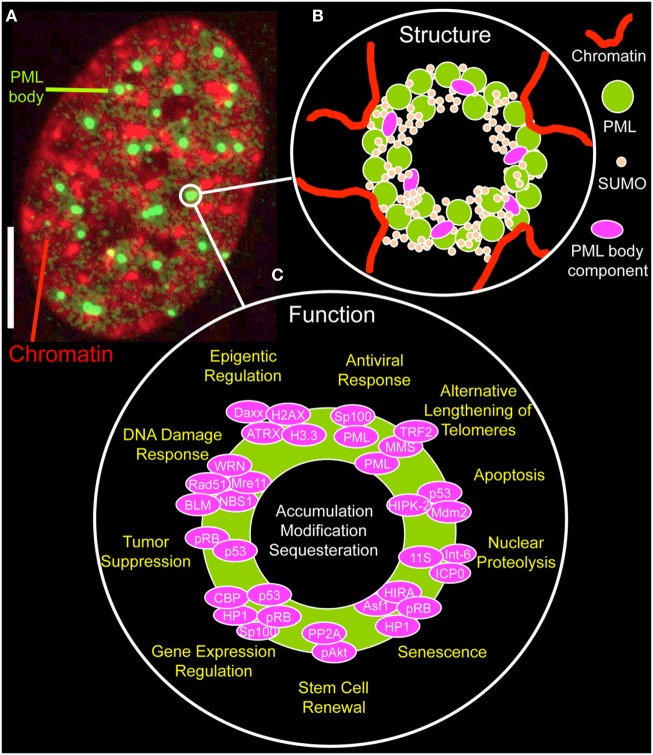
Structure and function of promyelocytic leukemia (PML) nuclear bodies. **(A)** Distribution of PML protein in a cell nucleus of a MRC-5 (primary human lung fibroblast) cell. The micrograph shows the immunofluorecence signal of an antibody directed to all PML isoforms (green, monoclonal antibody E-11, sc-377390, Santa Cruz Biotechnology, Heidelberg, Germany) along with DAPI fluorescence (red) of a mid-confocal section of the nucleus. Bar; 5 µm. **(B)** Structure of PML nuclear bodies. SUMOylated PML protein subunits are the building blocks of a shell-like structure in the periphery of the nuclear body. Additional PML body-interacting proteins may bind to PML, specifically to the C-termini of the various PML isoforms, to the poly-small ubiquitin-related modifier (poly-SUMO) chains or to SUMO-interaction motifs. PML nuclear bodies’ are in direct contact with chromatin fibers, which contribute to the bodies physical stability. See Figure 3 for more information on the assembly mechanism. **(C)** Proposed functions of PML nuclear bodies. Probably more than 100 proteins permanently or transiently bind to PML NBs. According to these protein’s function, many different physiological roles as depicted have been proposed for PML NBs.

By immunofluorescence light microscopy, normal PML bodies display as spherical structures, ranging in size from 0.2 µm up to 1 µm (Figure 2A). By electron or super-resolution light microscopy, PML protein is concentrated in a ~100 nm thick shell in the periphery of the nuclear bodies with no chromatin or RNA inside them (34–37). The shell also contains SUMO isoforms and other PML body components, such as SP100 (Figure 2B) (35). This structural arrangement provokes the question on the nature and biological function of the inner core of a PML NB. Within the nuclear body shell, PML’s branched SUMO chains stabilize protein complexes as a “molecular glue” (see next section). In functionally specialized PML bodies, such as in *alternative lengthening of telomeres-associated PML nuclear bodies* (APBs) and in *immunodeficiency, centromeric instability, and facial dysmorphy* (ICF) syndrome cells, the inner core of PML bodies contains chromatin, namely telomeric DNA in APBs or pericentric satellite heterochromatin of chromosome 1 in the giant PML bodies of ICF cells (38, 39).

The number of PML NBs varies between 5 and 30 depending on the cell-type, the cellular differentiation status, and the cell cycle. During interphase PML bodies are positionally stable through their physical and probably functional interplay with the surrounding chromatin (Figure 2B) (2). Yet, PML NBs are also dynamic structures that undergo significant changes in number, size, and position particularly in response to cellular stress (4). One example is fission of PML bodies into smaller bodies in early S phase (40, 41, 42). PML NBs may lose their structural integrity based on modifications or structural alterations in adjacent chromatin associated with DNA replication.

## PML Nuclear Body Function

Systems biological analyses based on online repositories, most notably the Nuclear Protein Database^1^ (43) have predicted, that more than 150 nuclear proteins have the ability to interact with PML bodies (44, 45). The “Biological General Repository for Interaction Datasets” (BioGRID) lists 243 unique protein interactions.^2^ Resident factors of PML NBs include, beside all PML isoforms, SUMO paralogs, Daxx, and SP100 (46). Most other factors only transiently accumulate at PML bodies under specific stress conditions or in specialized PML bodies, such as APBs or the giant PML bodies in ICF cells (39). In addition to the telomeric chromatin and shelterin core components, APBs accumulate DNA recombination and repair factors such as the MRN complex, RAD (*rad*iation sensitivity) family members, RPA and WRN (38, 47).

The functional diversity of transient PML NB components is likely the basis of the many different biological roles ascribed to these nuclear structures (Figure 2C) (5, 48). PML NBs have been functionally linked to apoptosis (49), nuclear proteolysis (50), senescence (51), stem cell renewal (52, 53), regulation of gene expression (54), tumor suppression (55), the DNA damage response (40, 41, 56), telomere elongation and stability (47, 57), epigenetic regulation (37, 58), and antiviral responses (59) (Figure 2C). Not surprisingly, functional annotation of PML nuclear body proteins show an enrichment of terms related to cell cycle control, cellular stress response, DNA repair, and protein modification processes (44). More globally, the various aspects of PML NB functions mainly point to their role in genome maintenance (18).

One hypothesis for the integration of all of these functions in a unifying concept is based on the idea that PML NBs provide a stable protein scaffold onto which binding partners associate for their efficient PTM or sequestration (Figure 2C) (28, 60, 61). Controlled accumulation at or release of specific nuclear factors from the nuclear bodies may enhance their functional interaction based on mass-law action, thereby fine-tuning signaling cascades through the nucleoplasm. This mechanism may enable chemical reactions or complex formation between low-abundance nuclear factors, as was also suggested for other subnuclear structures such as Cajal bodies or nucleoli (62). More specifically, PML NBs may be SUMOylation hot spots. This hypothesis is driven by the observation that most components of the SUMOylation machinery concentrate in PML NBs (45).

## PML Nuclear Body Assembly

**Figure 3:**
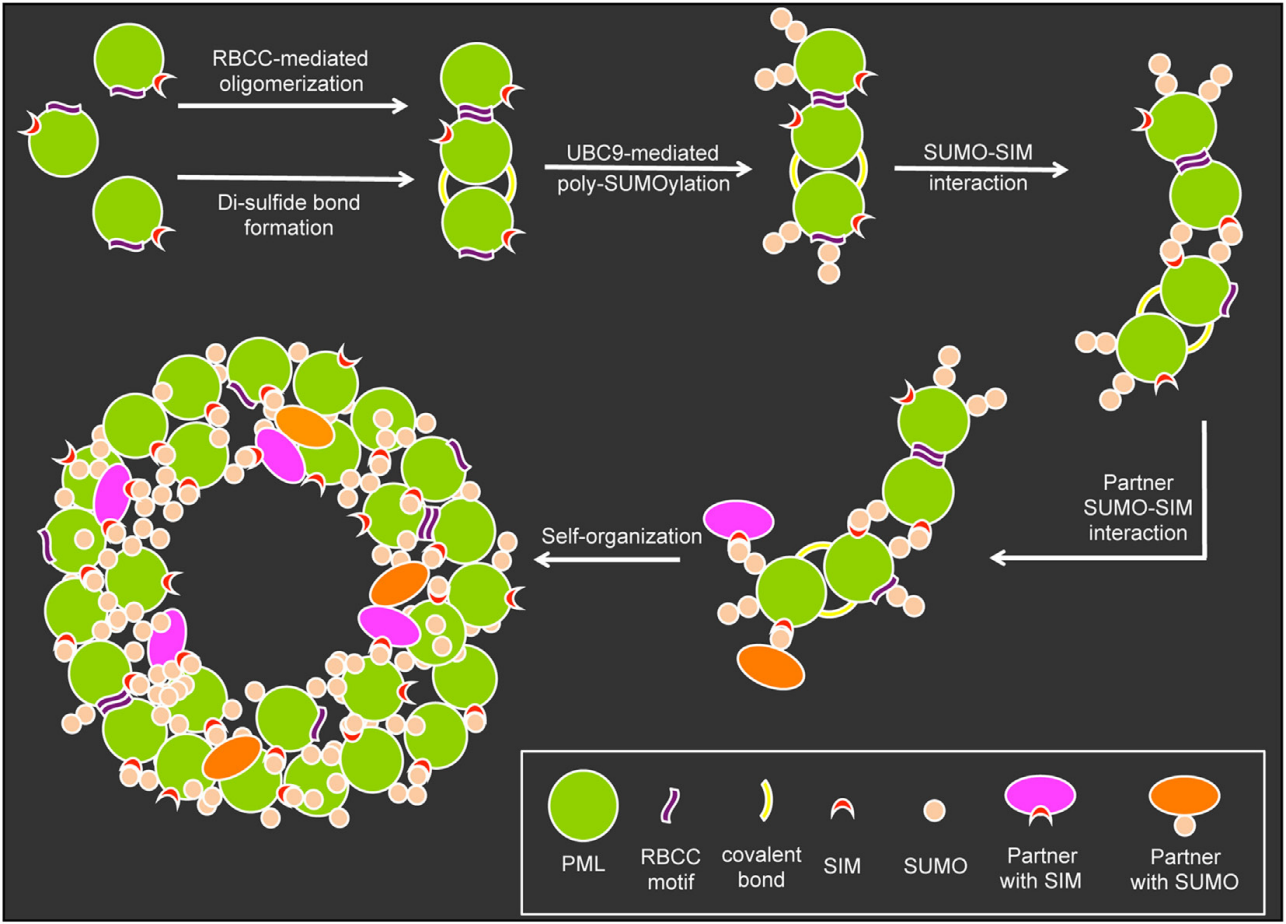
Assembly of promyelocytic leukemia (PML) nuclear bodies. The assembly of PML nuclear bodies is initiated by oligomerization of non-SUMOylated PML monomers. Oligomerization occurs *via* weak non-covalent interactions through the RBCC motif and covalent di-sulfide bonds between cystein residues. The E2-small ubiquitin-related modifier (SUMO) ligase UBC9 then promotes (poly-)SUMOylation of the PML moieties which allows for multiple SUMO–SUMO interacting motifs (SIM) interaction possibilities to form larger aggregates. Binding partners carrying SIMs and or SUMO residues can bind to the preassembled aggregates to form a normal PML body based on self-organization.

The formation and structural integrity of PML NBs relies on at least five basic mechanistic principles: (i) oxidation-driven intermolecular disulfide cross-linking of PML, (ii) the self-oligomerizing properties of PML’s RBCC motif, (iii) the poly-SUMO chains on the three major target lysines, (iv) the non-covalent interaction of SUMO with SUMO interacting motifs (SIM) in nuclear body-associated factors, and (v) specific sequences in various PML protein isoforms (Figure 3). In the initial step of nuclear body assembly, oxidized PML monomers allow the formation of disulfide-crosslinked covalent multimers that self-organize into the NB outer shell (9, 63). Non-covalent homodimerization mediated by the RBCC domain may be similarly important for the early PML NB assembly step, since the isolated RING domain of PML very efficiently forms multimeres *in vitro* (64). Subsequently, UBC9-mediated poly-SUMOylation, SUMO/SIM interactions (9, 65) and addition of SUMO and/or SIM-containing binding partners create a mature PML body with a peripheral scaffold consisting of the six different PML isoforms, their SIM motifs and the poly-SUMO chains (Figure 3). Recently, it was demonstrated that certain regions in the C-terminal domains of specific PML isoforms are also important for NB assembly and function (32, 33). These findings add an additional layer of complexity in the structural and functional maintenance of PML NB integrity. The PML nuclear body scaffold offers a multitude of potential sites to which an assortment of PML-interacting, SIM-containing, and/or SUMOylated partner proteins may bind transiently to a more or less extent. The varying residence times (Rts) of binding partners at PML NBs would be expected to depend on the number and strength of their individual interaction modules (66). This is in line with the presence of several SUMOylation sites and SIMs in major PML-NB components including PML, SP100, DAXX, HIPK2, UBC9, PIASy, and RNF4 (9).

The iterative nature of the multiple binding sites creates a multivalency, which has now been suggested to be responsible for the compartmentalization activity of PML NBs through the biophysical mechanism of phase-separation (67). Although only inferred as probable from GFP-SUMO/RFP-SIM phase-separation data obtained *in vitro*, the Banani et al. report suggests that that the polySUMO/polySIM interfaces in PML NBs may form phase-separated liquid droplet structures in living cells (68). Thus PML NBs belong to the family of viscous liquid, membrane less nuclear compartments, which may function as phase separating condensates equivalent to lipid droplets (69). The biochemical environment within a phase-separating PML body is different from that in the surrounding nucleoplasm, and this difference could enable unique strategies for regulating nuclear response pathways, including (a) regulation of enzyme reaction kinetics (i.e., posttranslational modifications), (b) regulation of the specificity of biochemical reactions, (c) sequestration of molecules, and (d) buffering cellular concentration of molecules (67).

Cell cycle-dependent disassembly of PML NBs begins upon de-SUMOylation of PML at the onset of mitosis. The spherical shell structure of PML NBs breaks down and other NB components such as SUMO, SP100, and DAXX detach or are removed. During mitosis PML aggregates into so-called mitotic accumulations of PML protein (MAPPs) (40, 41). Interestingly, PML bodies form stable interactions with early endosomes throughout mitosis and the two compartments dissociate in the cytoplasm of newly divided daughter cells (70). When followed through the telophase/G1 transition, Chen et al. observed that GFP-tagged MAPPs become trapped in the newly formed nuclei but also that many PML NBs are formed *de novo* at different sites in daughter nuclei. This suggests that PML NBs can assemble from both, MAPPs as well as soluble PML monomers in G1 (71). At the M/G1 border of the cell cycle, MAPPs also complex with FG repeat-containing peripheral components of the nuclear pore complex to become CyPNs (cytoplasmic assemblies of PML and nucleoporins) (72). Within CyPNs, PML appears to be instrumental in a novel, nuclear pore-independent, mechanism of nucleoporin and nuclear cargo protein targeting to the reforming G1 cell nucleus (73). The recruitment of SP100 and DAXX into newly formed PML NBs occurs considerably (ca. 30 min) later than PML NB genesis itself, suggesting that tightly controlled PTMs are required for full maturation of functional PML bodies in early G1 (71).

## PML in Tumorigenesis

So far, we have summarized some aspects of PML NB biology derived from microscopic, cell, and molecular biology approaches. Another branch of PML research has tackled questions on PML protein function by means of genetics. These approaches uncovered PML’s role in cancer biology. *PML* was originally identified as a potential gene of interest in tumorigenesis due to its association with acute promyelocytic leukemia (APL). APL is a rare but aggressive subtype of white blood cell cancer, characterized by an accumulation of promyelocytes in the bone marrow and peripheral blood (74). The majority of APL patients are characterized by the t(15;17) chromosomal translocation that reciprocally joins the *PML* and *retinoic acid receptor* α (*RAR*α) genes, resulting in balanced expression of PML-RARα and RARα-PML fusion proteins (1). While PML-RARα blocks differentiation of promyelocytes by suppressing the transcriptional function of RARα, PML-RARα disrupts the structure of PML nuclear bodies through formation of PML-RARα/PML heterodimers. This phenotype was observed in 99% of APL patients (75). Treatment of APL for many years was retinoic acid, arsenic trioxide or a combination of the two, which, fortunately, seemed to cure most APL patients. It is now known that the mechanism of this therapy involves targeting of the PML/RARα fusion protein to proteasomal degradation (76). Strikingly, drug treatment reverses the pathological microspeckled PML distribution in the nucleus of APL cells toward the regular morphology of PML nuclear bodies (77).

Another link between cancer and *PML* became evident by comparing PML protein expression in normal and neoplastic human tissues. Such studies documented loss of PML expression in breast carcinoma (78), gastric cancer (79), small cell lung carcinoma (80), and in invasive epithelial tumors (81). Furthermore, microarray analyses of PML mRNA expression showed complete loss of or strongly reduced PML transcript expression in many different human neoplasms, including colon, prostate, and breast adenocarcinomas, as well as in lung, CNS, germ cell, and non-Hodgkin’s tumors/lymphomas (8). The same study reported that PML protein is also frequently overexpressed in carcinomas of larynx and thyroid, epithelial thymomas, Kaposi’s sarcoma, and in Hodgkin cells, a tumor of cytokine-producing cells. The latter phenomenon may be attributable to strong upregulation of the PML gene after Interferon induction (82). Taken together, loss of expression in many (but not all) cancer types have suggested that PML works as a tumor suppressor (83).

## Tumor Suppressor and Oncogenic Functions of PML

Beside the correlative connection between carcinogenesis and PML expression, there is plenty of experimental evidence for a direct tumor-suppressive role of PML. Several independent studies have demonstrated that overexpression of PML can slow down or block cell cycle progression in a variety of cancer cell lines (6, 81, 84). Likewise, in primary human or mouse fibroblasts overexpression of PML isoform IV induces a stable senescence-associated cell cycle arrest (85, 86). Further analyses of typical stress-response pathways revealed the involvement of the tumor suppressors pRb and p53 in PML overexpression-induced cellular senescence (86, 87). However, the molecular details of PML action along the pRB and/or p53 tumor suppressive pathways remain elusive. Besides in cellular senescence, PML has an essential functional role in apoptosis (49). This is based on initial observations on the first reported PML knock-out mouse model, where splenic lymphocytes and thymocytes from Pml^−/−^ mice show barely half the capacity of wild-type cells to initiate apoptosis after ionizing radiation or after induction of the cytokine death-receptor pathway (88). As already pointed out, PML loss correlates with the progression of many cancers and in most cases low PML expression is associated with poor prognosis. The tumor suppressor function of PML NBs may be linked to their ability to accumulate many proteins involved in DNA damage response and repair pathways, which is believed to stabilize DNA repair complexes and enhance their activities (4, 13, 60). In support of this hypothesis, it was shown recently in a knock-in mouse model, that intact PML bodies are critical for DNA damage responses. Functional assays in mice expressing PML but lacking PML NBs showed impaired homologous recombination (HR) and non-homologous end-joining repair pathways, with defective localization of Brca1 and Rad51 to sites of DNA damage (89). Thus, although the physiological function of PML and the nuclear bodies have not been thoroughly elucidated, their tumor-suppressive role by supporting DNA damage response pathways may be common to all of these potential functions (19, 89).

The lack of PML is not necessarily a tumor-promoting event. Functional analysis of the hematopoietic stem cell compartment in mice have uncovered that PML is required for leukemia initiating cell maintenance (90). The authors suggest a new therapeutic approach for eradication of cancer-initiating cells in leukemia through pharamacological inhibition of PML. This and other reports have lead to the suggestion that PML may act as both a tumor suppressor and an oncogene, depending on the cellular context (91). Along these lines it was also demonstrated that PML targeting impacts on breast cancer (BCa)-initiating cell function, and hence on cancer initiation and dissemination in BCa (92). Furthermore, in triple-negative breast cancer cell and mouse models PML promotes cell migration, invasion, and metastasis through binding to regulatory regions of HIF1A target genes (93). These initially unexpected findings clearly suggest a previously underestimated importance of PML in the maintenance of some tumors.

## Laser-Based Fluorescence Imaging and Manipulation Approaches to Analyze Nuclear Protein Function

Inter- and intracellular mechanisms of molecular communication may be better understood through direct visualization. In the past decades, advancements in imaging technologies have expanded our ability to access and analyze in living specimen the morphology of tissues and cellular components. These enabled analyses of fine-structural features at the nanoscale level, precise localization, and the dynamic interplay of single and macromolecular assemblies that drive cell growth, the cell cycle, differentiation, and cell death (20–37). Super-resolution light microscopy delivered images with unprecedented sensitivity and clarity allowing the exploration of interactions between individual molecules with a distance resolution as low as 20 nm (94). New fluorescence fluctuation microscopy (FFM) approaches provided the basis for determining the biophysical and interaction properties of single molecules in living cells (95, 96). Laser-based FFM analysis tools are outlined below but many more exist, all of which unfortunately cannot be covered by this overview, including single particle tracking (SPT), light sheet microscopy, total internal reflection microscopy just to name a few. To acquire the full picture of live cell laser-based imaging technologies, we refer to recent excellent reviews on these topics (20, 97–99). Altogether, a plethora of new live cell imaging techniques have been developed which even large research groups are unable to establish to a broad extent in their departments. To address this, dedicated advanced light microscopy imaging facilities are extremely helpful as their members are usually microscope experts (100). However, we believe that research laboratories are still reluctant in exploiting the full potential of microscopy facilities. We therefore provide an introductory overview on some imaging instrumentation which are covered by such facilities and provide specific examples in PML biology to encourage cancer cell biologists and biochemists to extend their experimental approaches toward the exciting new imaging technologies.

## Fluorescence Recovery after Photobleaching (FRAP)

Arguably, one of the most commonly used approaches to study dynamic cellular processes in living cells is FRAP (101). FRAP is able to access average dynamics of diffusing molecules within the observation volume. The original description of FRAP was coined continuous fluorescence microphotolysis, which itself has been established for more than three decades (102). When subjected to repeated cycles of excitation and emission, fluorescent molecules eventually lose their ability to emit fluorescence, enabling the creation of photobleached spots of fluorescent molecules in solution or in living cells by the application of a laser beam. By monitoring the redistribution of the fluorescent molecules from the unbleached volume, their diffusion or transport properties can be assessed (101). FRAP and related techniques such as point continuous photobleaching, fluorescence loss in photobleaching, inverse-FRAP, and photoactivation/conversion have been developed in the past, each suitable to quantitatively assess specific biophysical properties of the molecule under investigation (97). However, the limitations and pitfalls of FRAP experiments, in particular when they are employed to extract biophysical parameters also, have to be considered. Things to consider include the complete set-up of the FRAP experiment (103), knowledge on the bleach volume profile (104), as well as the potential phototoxic effects elicited by the bleaching laser beam (105).

**Figure 4:**
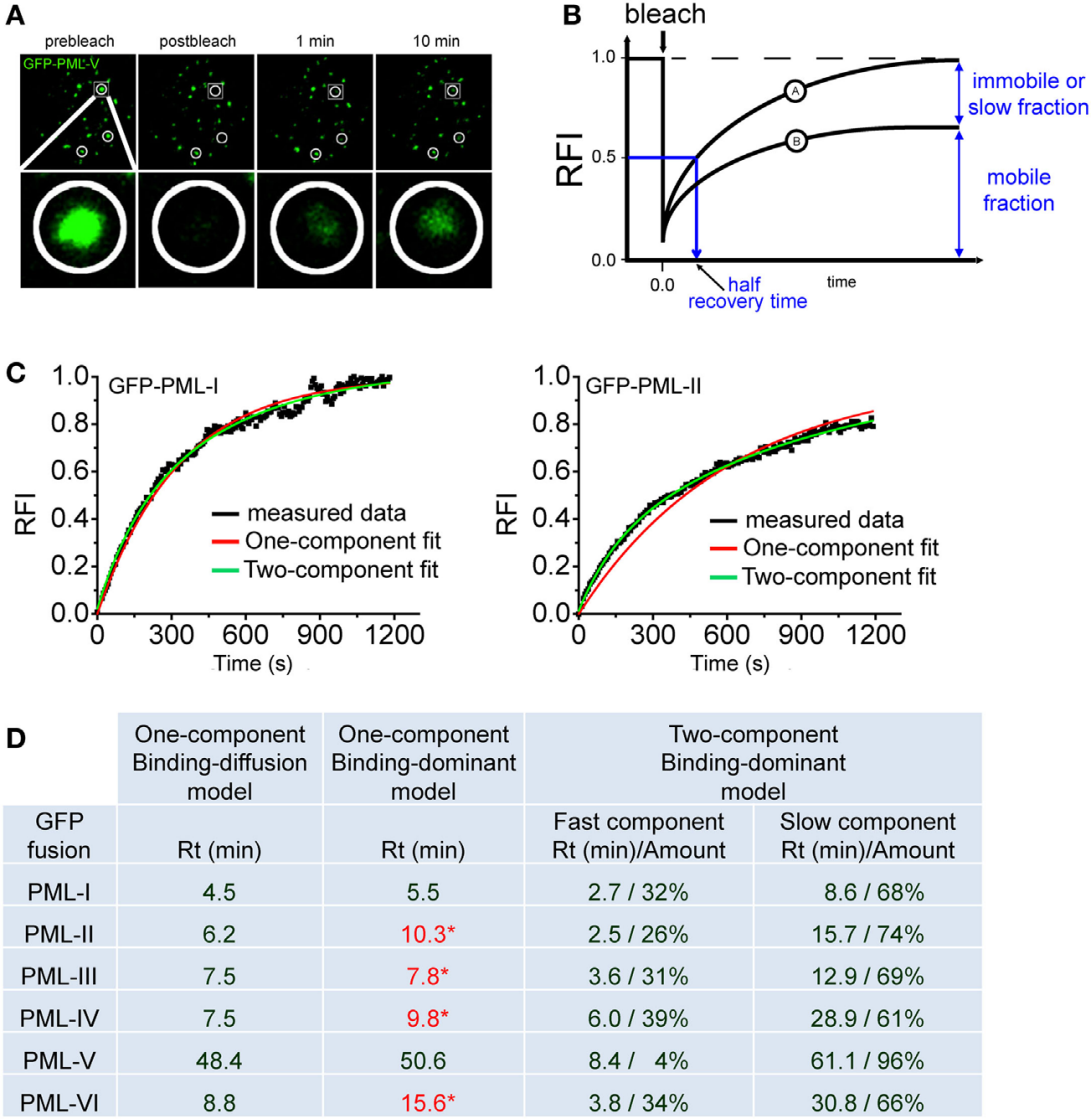
Fluorescence recovery after photobleaching (FRAP) to assess component exchange at promyelocytic leukemia (PML) nuclear bodies. **(A)** A typical FRAP experiment is shown. Two circular regions in the nucleus of a GFP-PML-V expressing U2OS cell were exposed to a short 488 nm laser bleach pulse and fluorescence redistribution was monitored over time. A third circled unbleached region at the bottom of this nucleus is shown as a positive control. One particular bleach spot is shown in a magnified view in the bottom panels. **(B)** Quantitation of FRAP experiments. After background subtraction, compensation for imaging-induced photobleaching and normalization, typical FRAP curves are obtained. FRAP curve A shows full recovery to prebleach fluorescence values indicating complete exchange of the GFP-tagged protein in the bleached spot within the observation time. In FRAP curve B, fluorescence recovery is not complete within observation time indicating an immobile fraction of molecules or a fraction with a very slow exchange rate. **(C)** FRAP curve fitting using exponential functions. FRAP curves for GFP-tagged PML-I (left) and PML-II (right) were fitted with one-component (red) or two-component (green) exponential functions. **(D)** Table showing the residence time (Rt) in minutes of GFP-tagged PML isoforms I to VI derived from fitting FRAP curves employing different mathematical models (See text for details). * numbers in red letters represent Rt values derived from one component modeling which failed to precisely fit to the measured FRAP curve as shown for GFP-PML-II in **(C)**.

Figure 4A shows a typical FRAP experiment for GFP-tagged PML (isofom V) at nuclear bodies. Measuring the redistribution of fluorescence into the bleached region then yields the FRAP recovery curve (Figure 4B). During the observation time of the FRAP experiment, the fluorescence in the bleached region may return to the prebleach value (Figure 4B, curve A) or not (Figure 4B, curve B). Incomplete recovery even after long observation (>1 h) has been observed for some chromatin-binding proteins, which suggests the presence of immobile or very slow exchanging populations of molecules (106, 107). FRAP data can be analyzed using mathematical models to yield kinetic parameters (108). Today, many FRAP models of processes in the cell nucleus assume that the proteins undergo diffusion as well as binding/unbinding events at chromatin or other more static subnuclear structures such as nuclear bodies. Importantly, both diffusion and binding/unbinding events contribute to the spatial dynamics of nuclear proteins (109, 110).

With respect to PML protein exchange at nuclear bodies it is safe to assume a binding-dominant behavior because of the very slow exchange rates as observed by FRAP (Figure 4A). Previously, the residence time (Rt) at nuclear bodies of all PML isoforms had been determined by FRAP using a binding-diffusion model based on differential equations (29, 66). It was therefore interesting to compare different modeling approaches. Figure 4C shows two examples of fitting FRAP data obtained for GFP-tagged PML isoforms I and II. Interestingly both, one- and two-exponential functions delivered good fits to the FRAP curve for GFP-PML-I but not for GFP-PML-II, where only a two-component exponential function gave good fit results (Figure 4C).

To obtain a complete picture we collected the Rts of all PML isoforms after fitting to one- and two-component exponential functions (Figure 4D). This approach delivers the Rt of the protein under investigation (110). One-component exponential fits were successful for GFP-tagged PML-I and PML-V, while FRAP curves for the other isoforms could only be fitted with two-component exponential fits (Figure 4C and data not shown). For comparison, the table includes the data we previously obtained by application of a binding-diffusion model based on more sophisticated differential equation modeling to analyze the FRAP curves (66). The table shows that the Rts of PML isoforms at nuclear bodies as deduced from one-component exponential fits, is convincingly close to those obtained from assuming a binding-diffusion model (Figure 4D) although the fits are not satisfactory for PML-II, -III, -IV, and -VI (values in red letters). In particular, the very long Rt of PML-V (~50 min) is confirmed. This observation is fully consistent with the presence of a strong homo-dimerization domain we found in the unique C-terminus of PML isofom V (32). Obviously, this domain in PML-V confers additional binding strength toward PML bodies. Fitting with two-component exponential functions assumes the presence of two populations of molecules exchanging at PML bodies with different on/off rates. These functions provided perfect fits for all PML isoforms (Figure 4C, green curves, and data not shown), and the Rts are shown in Figure 4D. Interestingly, the two-component fits deliver considerably large populations of PML isoforms IV (61%) and VI (66%) with a Rt of ~half an hour (Figure 4D). This suggests that a subfraction of these isoforms may contribute to the structural integrity of nuclear bodies through stable incorporation.

In conclusion, Figure 4 suggests that different FRAP modeling approaches, despite subtle differences, arrive at overall similar Rts for PML isoforms at nuclear bodies. It should be noted however that these long Rts do not necessarily reflect the time in which one PML molecule stays bound to one and the same specific binding site. Long Rts may also originate from PML molecules undergoing rapid binding and unbinding events at multiple adjacent binding sites (in our case at the nuclear body) without leaving the observation volume (110). If binding/unbinding events do not occur on well-separated time scales, the interaction parameters may not be readily extractable from the FRAP curves (111). A combination of different FFM approaches may be required for accurate determination of binding parameters (112). To assess binding/unbinding events at PML NBs at higher resolution, the tool kit should be extended to single particle tracking (SPT) since this approach is able to quantitatively describe several populations of molecules with distinct binding properties (113).

## Fluorescence (Cross) Correlation Spectroscopy

**Figure 5:**
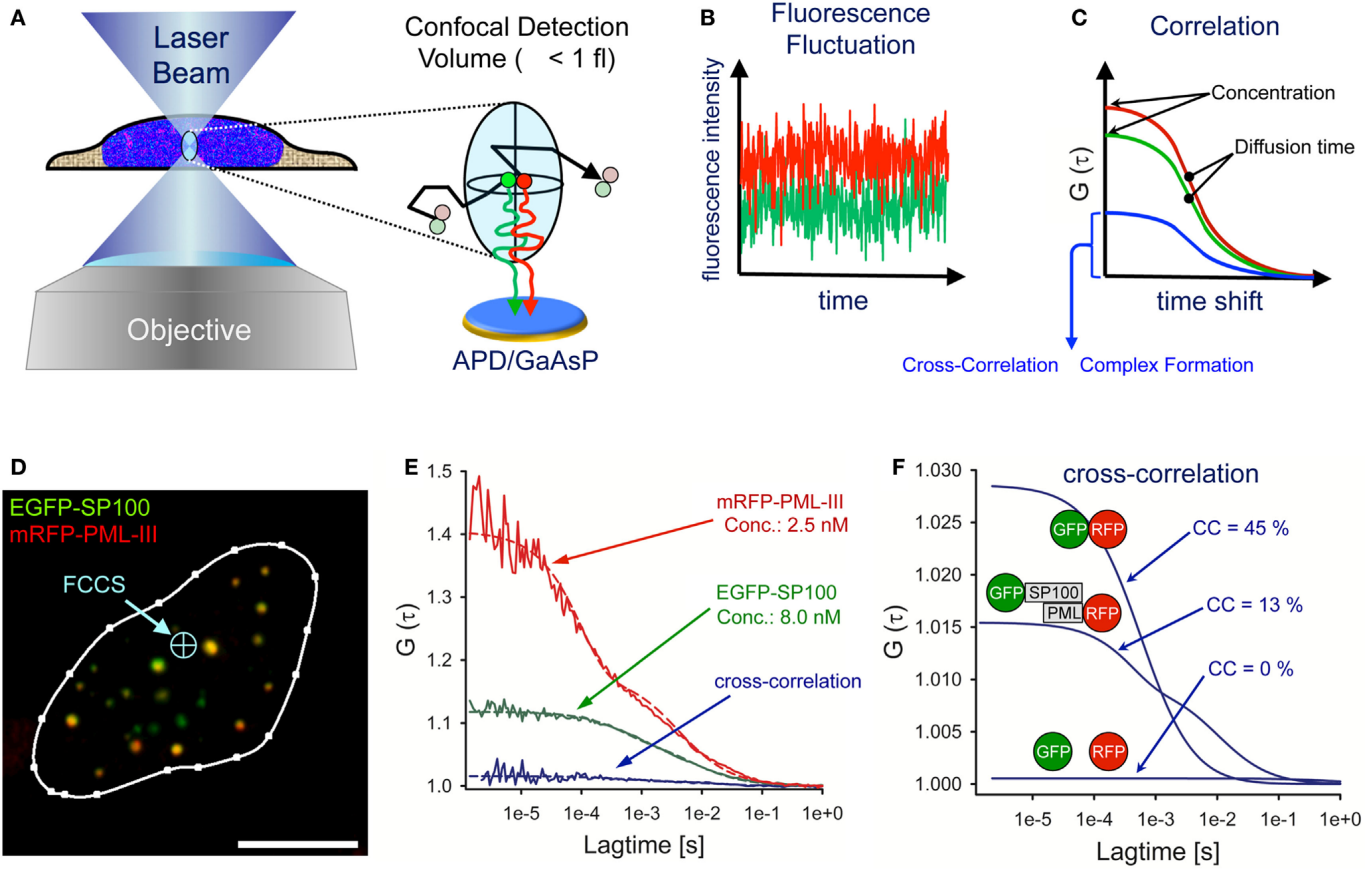
Fluorescence cross-correlation spectroscopy (FCCS) analysis of promyelocytic leukemia (PML) nuclear body components. **(A)** Schematic side view of a living cell with the FCS laser beam focused to a position within the nucleus. The objective creates a laser light-illuminated subfemtoliter measuring volume in which single fluorescent molecules are excited to emit photons. The photons are counted on an avalanche photodiode (APD) or a galliumarsenidphosphid (GaAsP) detector as a time series of fluorescence intensity **(B)**. These statistical fluctuations are mathematically processed using an autocorrelation algorithm, from which biophysical parameters such as the particle concentration, the diffusion coefficient and complex formation properties can readily be assessed **(C)**. **(D)** Confocal live cell image of a U2OS nucleus coexpressing EGFP-SP100 and mRFP-PML-III (bar: 5 µm). The FCCS laser beam (light-blue) can be positioned anywhere in the cell. **(E)** By fitting the measured FCS data points (solid lines) to appropriate diffusion models (dashed lines), one can extract from the reciprocal of the amplitude and the decay half-time value the number of particles in the detection volume (*concentration*) and the *diffusion time*, respectively. The cross-correlation (CC) result of EGFP-SP100 and mRFP-PML-III are also shown. **(F)** CC results in the nucleus of living cells for a GFP–RFP fusion protein (positive control, high CC), GFP and RFP as individual proteins (negative control, no CC) and the measurement performed in **(D,E)**.

Fluorescence correlation spectroscopy (FCS) is an *in vivo* method that analyses diffusing particles in a diffraction-limited illumination ellipsoid (114, 115). The FCS detection volume is created by a laser beam in a pinhole-adjustable confocal optical system focused through a high numerical aperture objective (Figure 5A). The FCS detection volume is defined by the point spread function of the objective and the confocal pinhole. The excitation laser beam determines how much of the detection volume is excited and the final observation volume is determined by the overlap of excitation and detection volumes. For objectives with a high numerical aperture (i.e., NA = 1.4) the effective measuring volume is ~1 fl (116). Photons emitted from diffusing fluorescent particles are counted continuously over time through the same optics using sensitive avalanche photodiodes (APDs) or galliumarsenidephosphide (GaAsP) hybrid detectors at single molecule resolution (Figure 5A) (117). The fluorescence intensity fluctuations are recorded over time (Figure 5B). Particle concentration is reflected by the fluctuation amplitude, whereas the frequency gives information on the diffusion times of the fluorescent particles. For quantitative evaluation, the photon trace is correlated with a time-shifted replica of itself (autocorrelation) at different time values (Figure 5C). The amplitude of the autocorrelation curve is inversely proportional to the average number of fluorescent molecules in the confocal volume allowing determination of particle concentration (Figure 5C). A more detailed overview on the theory, history, and application of FCS can be found here: (118, 119).

In fluorescence cross-correlation spectroscopy (FCCS), two spectrally distinct fluorophores are measured in the same detection volume at the same time (Figures 5A,B, red and green lines) and correlated by cross-correlation (CC) (Figure 5C, blue curve). The amplitude of the CC curve is directly proportional to the degree of complex formation and/or direct interaction between the two fluorescent particles (120). A practical guide to set up FCS and FCCS experiments in living cells can be found here (121–123).

An example of FCCS measurements of PML body components is shown in Figure 5D. The image shows a live-cell confocal snapshot of a U2OS cell nucleus transiently expressing EGFP-SP100 and mRFP-PML III. The FCS laser spot was parked at a position in the nucleoplasm where the fluorescence signals of the fusion proteins are extremely low (Figure 5D, blue arrow). EGFP and mRFP fluorescence fluctuation was then recorded over time (10 × 10 s measurements) and the fluctuation data correlated for each fluorophore (Figure 5E). By fitting theoretical model functions to the measured autocorrelation curves, the diffusion coefficient and the concentration of the diffusing species can be extracted. In this particular cell nucleus (Figure 5D), the concentration of EGFP-SP100 and mRFP-PML-III was 8.0 nM and 2.5 nM, respectively, demonstrating the power of FCS to work at extremely low expression levels (Figure 5E). The diffusion coefficient in the nucleoplasm outside nuclear bodies for these PML body components had been determined previously (*D*_SP100_ = 1.23 µm^2^ s^−1^, *D*_PML-III_ = 1.63 µm^2^ s^−1^) (66).

Cross-correlation analysis between EGFP-SP100 and mRFP-PML-III revealed a small but significant amplitude above the value of 1.0 (Figure 5E, blue curve indicated with GFP-SP100 and RFP-PML), indicating the formation of complexes between these fusion proteins. To evaluate this observation, the CC was compared with values obtained for individually expressed GFP and RFP molecules (negative control) as well as a GFP-RFP fusion protein (positive control) (Figures 5E,F). Experiments with these fluorochromes determine the dynamic range of the FCCS set-up. The mathematical delineation of the CC values is described elsewhere (124). Analyzing EGFP and mRFP as single molecules in our system resulted in CC = 1.001, indicating 0% complex formation while for the mRFP-EGFP fusion protein we observed a CC amplitude of 1.029, corresponding to 45% complex formation. The CC value for EGFP-SP100 and mRFP-PML-III was CC = 1.010, indicating that in this cell nucleus ca. 13% of SP100 molecules reside in a complex with PML (Figures 5E,F). These analyses demonstrate that FCS and FCCS, although technically somewhat more demanding than for example FRAP experiments, provide an extremely powerful tool to precisely extract biophysical and interaction data on diffusing molecules of interest in living cells.

## Raster Image Correlation Spectroscopy (RICS)

Ideally, in the assessment of biophysical parameters of mobile molecules in living specimens, one wants to know the space-resolved behavior of single molecules in terms of their kinetics and interactions and without the disturbance of the equilibrium state, as occurs in FRAP. All of these parameters are provided by RICS (125). Data acquisition in RICS is quite simple as only a 2D confocal image or time series analysis is required. The scanning encodes dynamic information within a single image, which can then be extracted using RICS. The processing of the resulting images, however, is not trivial: RICS data are computed from the power spectrum of the spatial autocorrelation function that is obtained from the intensity images by 2D fast Fourier transformation algorithms (125).

Raster image correlation spectroscopy thereby expands the accessible timescales of FCS as it can resolve dynamics in the range of microseconds to seconds with still a sufficient spatial resolution (125). Data in cells are most conveniently acquired as a time series stack by raster scanning of images of selected cell areas. Due to its broad dynamic access by analyzing the fluctuations between neighboring pixels in the *x*- and *y*-direction, nearly all diffusion processes that take place in cellular subregions can be studied (125). A major advantage of the RICS technology is that it can be used in principle on any commercial confocal microscope with analog detection (126). The software and application tutorials developed by the Enrico Gratton lab can be found as downloads here.^3^

**Figure 6:**
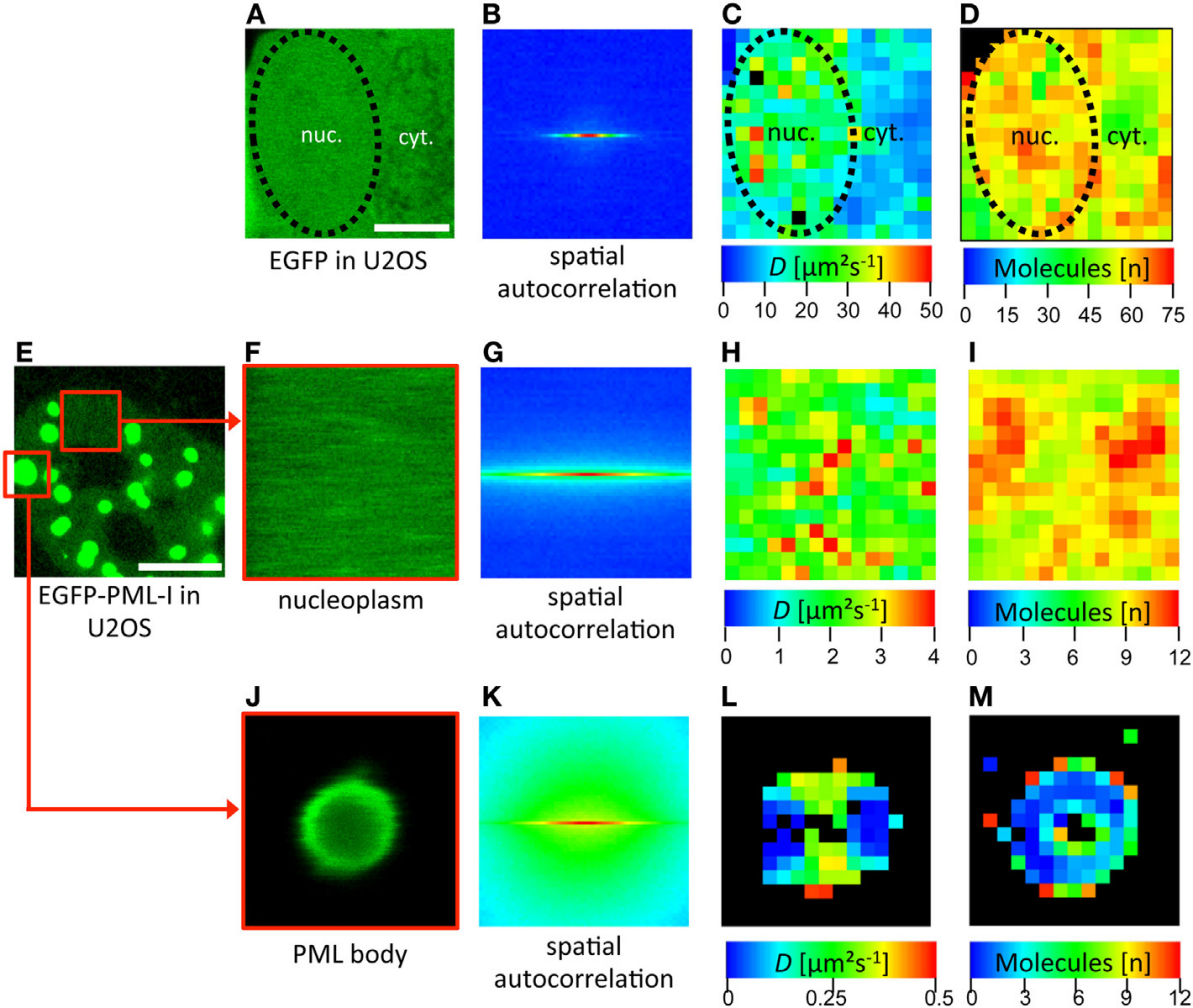
Spatial mapping of promyelocytic leukemia (PML) protein mobility in the nucleus by raster image correlation spectroscopy (RICS). **(A–D)** As an introductory example, RICS was performed in a U2OS cell expressing EGFP alone. **(A)** Shows a subregion of the cell containing nuclear (nuc.) and cytoplasmic (cyt.) parts. The nuclear outshape is indicated by a black dashed line. For RICS analysis, a time series of GFP fluorescence images was acquired by confocal microscopy. Thus, the fluorescence intensity of each pixel is collected and the spatial autocorrelation is obtained per image **(B)**. The image stack serves to increase the SNR and to be able to remove immobile and slow molecules. This analysis generates spatial maps of the diffusion coefficient **(C)** and the number of free molecules which contributed to the assessment **(D)**. Since the shape of the spatial autocorrelation image indicates mostly freely diffusing species, the RICS data were fitted with a one-component 3D-free diffusion model yielding diffusion coefficients for EGFP in the nucleus between 10 µm^2^ s^−1^ and 50 µm^2^ s^−1^ **(C)**. RICS was then performed similarly in a EGFP-PML-I-expressing U2OS cell **(E–M)**. In the nucleoplasm, the diffusion coefficient of EGFP-tagged PML-I ranged between 1 µm^2^ s^−1^ and 4 µm^2^ s^−1^ **(H)**. RICS in a region containing a large PML NB **(J)** still delivered acceptable spatial autocorrelation quality **(K)**. This approach revealed a diffusion coefficient of EGFP-PML-I in or at PML bodies which was one order of magnitude lower than in the nucleoplasm **(L)**. Bars; 5 µm.

To measure RICS we have used a Zeiss LSM710 which conveniently provides a built-in RICS module in the ZEN microscope software. Some examples of RICS measurements are shown in Figure 6. As a positive control, a confocal time series was acquired in a subregion of a U2OS cell expressing EGFP (Figure 6A). RICS analysis then delivers a spatial correlation of this region (Figure 6B). By fitting of the correlation data with a 3D-free diffusion model, a spatially resolved diffusion coefficient map is generated (Figure 6C). This map shows that EGFP diffuses throughout the cellular volume with variable diffusion coefficients ranging between 5 µm^2^ s^−1^ and 50 µm^2^ s^−1^. The mean value for EGFP in the nucleus by RICS was 25 (± 5) μm^2^ s^−1^ (*n* = 12) consistent with FCS data (127). The RICS approach also delivers a map of the number of detected mobile molecules in the diffusion analysis (Figure 6D). RICS was then applied to a U2OS cell nucleus expressing EGFP-PML (isoform IV) (Figures 6E–M). Two subregions of the 2D confocal time stack were selected for RICS analysis. RICS analysis in the nucleoplasm (Figures 6F–I) showed that the diffusion coefficient of GFP-tagged PML-IV is about one order of magnitude smaller than that of GFP alone (Figure 6H), consistent with FCS measurements (66). This suggests incorporation of PML into larger complexes and/or interaction with an immobile structure (i.e., chromatin), or both. RICS can also be performed on large (but not small) PML nuclear bodies (Figures 6J–M). The resulting diffusion map reveals very slow diffusion of GFP-PML-IV at or within PML NBs (Figure 6L), suggesting that binding events predominante PML molecule mobility at or in the nuclear body.

The examples shown illustrate the power of RICS to determine spatial maps of concentrations, aggregation, diffusion and binding of mobile molecules in living cells using readily accessible instrumentation (125).

## Förster Resonance Energy Transfer (FRET)

The FRET process is a dipole–dipole interaction in which an excited donor fluorophore transfers energy to an acceptor molecule in nanometer vicinity without absorption and emission of a photon (128). FRET is therefore commonly employed to measure the spatial distance between two fluorophores in fixed as well as in living cells (129). The FRET efficiency depends on the distance between two adjacent fluorescent molecules. At the Förster radius distance between a FRET pair (typically around 5 nm), the FRET efficiency is 50% (130). This size regime is comparable to the size of many proteins, the distance within which proteins interact, and the distance between sites on multisubunit proteins. Therefore, FRET can deliver parameters on the distance between two distinct sites on a macromolecule, the distance between two fluorophore-tagged proteins, and hence if and how these two proteins interact (131). Basically five different FRET detection methods have been developed for light microscopy, including acceptor photobleaching, donor photobleaching, ratio imaging, sensitized emission, and fluorescence lifetime measurements (132).

**Figure 7:**
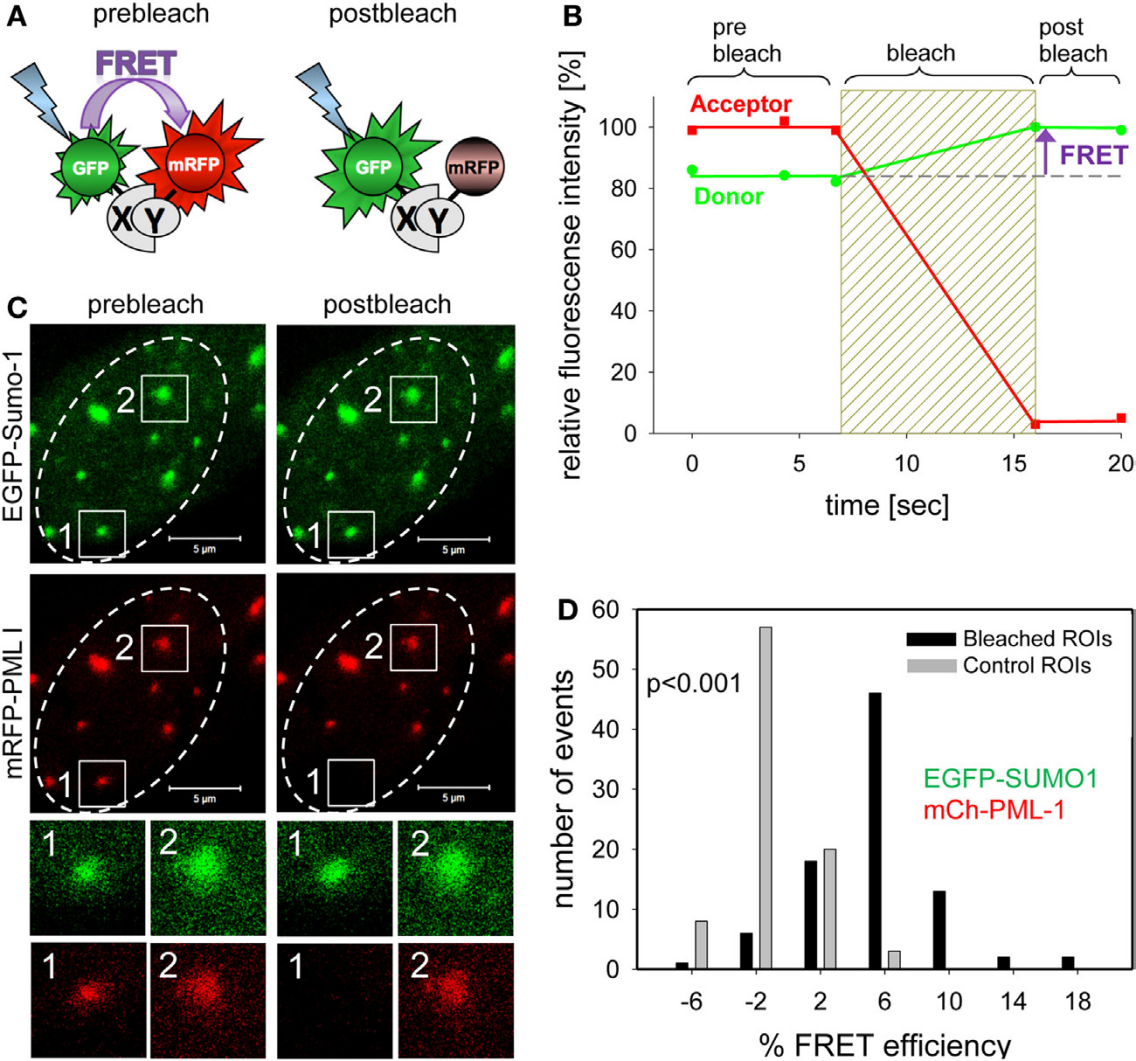
Complex formation assessment of promyelocytic leukemia (PML) body components by acceptor-photobleaching Förster resonance energy transfer (FRET). **(A)** Schematic explanation of acceptor-photobleaching Förster resonance energy transfer (abFRET). The energy donor EGFP and the energy acceptor mRFP are fused to proteins X and Y, which are sufficiently close (<10 nm) to each other to allow for FRET. Left side: the acceptor mRFP absorbs radiation-free energy from the exited donor EGFP resulting in decreased donor fluorescence intensity. Right side: acceptor mRFP is bleached and energy is no longer transferred from donor EGFP to acceptor mRFP resulting in an increase of donor fluorescence intensity. **(B)** Time courses of the fluorescence intensity of donor and acceptor during abFRET. Bleaching of the acceptor results in a fluorescence intensity increases of the donor indicating FRET (arrow). **(C)** Cell nucleus showing the location of EGFP-Sumo-1 and mRFP-PML I in PML-bodies. Two of them, spot1 and spot2, were selected for fluorescence intensity analysis before and after acceptor-photobleaching (see enlargements below). At spot 1, the acceptor fluorophore mRFP was bleached (compare prebleach and postbleach), whereas spot 2 was not bleached and served as control. **(D)** The donor fluorescence intensity variation observed during acceptor-photobleaching was determined for 89 not bleached control PML-bodies (see spot 2) yielding E_var_ (gray bars) and for 89 acceptor-photobleaching PML bodies (see spot 1) yielding E_FRET_ (black bars). The numbers of observed single cases (grouped into E_var_ or E_FRET_ value ranges of 4%) are displayed versus the values of E_var_ and E_FRET_.

In the past, we have mainly used acceptor photobleaching FRET (abFRET) to analyze spatial proximities within chromatin-interacting complexes (133). In abFRET, the acceptor chromophore is photobleached, thereby preventing FRET from the donor to the acceptor (Figure 7A). If the donor and acceptor were in sufficient proximity for energy transfer, photobleaching the acceptor results in an observable increase in donor fluorescence (Figure 7B). The measurement of abFRET only generates positive values when the distance between the donor and acceptor (in our case EGFP and mRFP, respectively) is between 3 and 8 nm. An abFRET example is shown in Figure 7C, where EGFP-Sumo-1 (green) and mRFP-PML I (red) are coexpressed in living cells. Two PML bodies were selected for analysis and acceptor photobleaching performed in region 1 but not in region 2 which served as internal control for non-FRET effects (Figure 7C). For quantitation, FRET efficiencies in bleached and unbleached regions are then plotted in a bar diagram (Figure 7D). The plot indicates that FRET between EGFP-SUMO-1 and mRFP-PML-I occurred in most of the cases (mean of FRET = 5.5%; black bars in Figure 7D), while the unbleached control spots show a FRET mean value of −1.4% (gray bars in Figure 7C). The FRET efficiency distribution is significantly different from that in control regions (*p* < 0.001, *n* = 88) (Figure 7D). Thus, Sumo-1 is in close proximity to PML I at PML NBs which was expected because of the covalent conjugation of SUMO-1 to PML in nuclear bodies (134). A similar abFRET approach has previously been used to document the functional interaction between the CHFR mitotic checkpoint protein and PML within PML NBs (135).

By expanding this abFRET approach, the individual spatial relationships between many PML NB components and probably even the degree of PML SUMOylation could now be determined to obtain a full picture of the molecular interaction landscape within PML NBs. This kind of approach proved successful in detecting the spatial inter-relationships within the large human kinetochore complex (136) as well as in smaller complexes such as the nucleosome (137). Thus, adding FRET techniques to the experimental tool kits in many laboratories would significantly increase the understanding of protein-protein interaction networks in cancer biology.

## Laser Microirradiation

**Figure 8:**
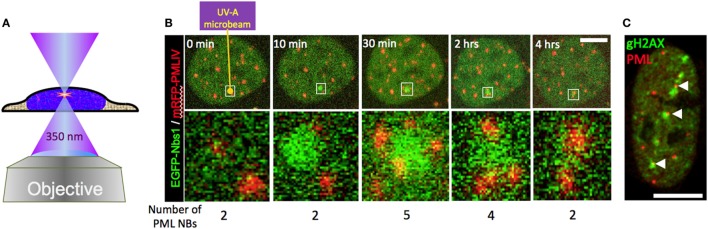
Behavior of promyelocytic leukemia (PML) bodies at UV-A microbeam induced DNA damage foci. **(A)** For laser damage induction, a pulsed 350 nm Nd:YLF (neodymium-doped yttrium lithium fluoride) UV-A laser (Spectra Physics) was coupled into a confocal laser scanning microscope (LSM 510) *via* the epifluorescence illumination path. The laser-microbeam is focused into the middle of the field of view by a 100×, NA 1.3 Plan Neofluar oil immersion objective (Zeiss). The Nd:YLF laser can be frequency-tripled delivering 20 ns duration pulses at 350 nm with user-defined energies from 1 µJ to 200 µJ at user defined repetition rates 1–1000 Hz. **(B)** A living U2OS cell expressing EGFP-tagged NBS1 and mRFP-tagged PML-IV was irradiated at a single defined spot with approx. 5 µJ of energy using the set-up described above (yellow arrow). Confocal stacks were acquired across the nucleus before and at indicated time points after the damage pulse. In **(C)**, U2OS cells were microirradiated at multiple positions with high laser power (40 µJ per site). Twenty-four hours after DNA damage induction cells were fixed and stained to detect the DNA damage marker gH2AX (green) and endogenous PML (red). Scale bars, 5 µm.

Experimental induction of DNA damage foci in living cells became an ideal method to analyze in time and space the recruitment and binding properties of repair factors at DNA lesions (138). Live cell imaging of DNA damage foci has been used to reveal the mobility of repair proteins, their assembly timing into repair sites, and the movement of damaged chromatin (139–141). DNA damage can be induced globally by ionizing radiation or radiomimetic drugs allowing for bulk analysis of the DNA damage response at multiple sites of DNA damage in the nucleus (142). Generation of single focal spots of DNA damage is instrumental to analyze single repair sites and became possible by targeted expression of endonucleases or microirradiation (143). Coupling of UV-A light-emitting lasers into confocal microscopes resulted in the development of laser-microirradiation technologies (Figure 8A) (144, 145). Laser lines in the visible range spectrum (405–514 nm) as well as multiphoton excitation (>750 nm) have been implemented in such devices (146). The advantages and disadvantages of the different laser systems to study cellular responses to DNA damage has been assessed (147). By fine-tuning the microirradiation system it is possible to discriminate between induction of base lesions, single–strand and double-strand DNA breaks. Special UV-suitable lenses (i.e., quartz glass) must be used to avoid energy loss and destroying the lenses. Objectives with a high numerical aperture should be employed to achieve diffraction-limited focusing and fine micromanipulation. A starting point to plan the application of microirradiation techniques can be found here: (148, 149).

The involvement of PML NBs in cellular DNA damage response/repair pathways became evident upon the demonstration of their colocalization with experimentally induced DNA damage foci (12, 13, 56, 150). UV laser microirradiation was also used in the initial studies to analyze in more detail the behavior of PML NBs in the vicinity of DNA damage in live cells (40, 41). These studies revealed intriguing morphological changes of PML NBs near the damaged chromatin, including moving toward the breaks, coalescing, and loss of positional stability. A recapitulation of these observations is shown in Figure 8B. In this experiment, a short pulse of 350 nm UV light was focused into the nucleus of a U2OS cell expressing GFP-tagged NBS1 (A DNA damage sensor protein) (green) and mRFP-tagged PML (red). As expected, NBS1 accumulates focally at the microirradiated chromatin spot within minutes. Shortly after, new PML bodies appear at the periphery of the damage focus (30 min). After 4 h, NBS1 has detached from the irradiated area and the number of PML NBs returned to preirradiation levels (Figure 8B, 4 h). The repair process has most likely been successfully completed by that time although direct evidence for successful repair is lacking. All these observations are consistent with previously reported data (40, 41). When DNA damage becomes irreparable, repair foci become permanent, as demonstrated for damaged telomeres (151). PML bodies stay stably associated with such irreparable DNA breaks (152). This phenomenon is illustrated in Figure 8C, where a U2OS cell was microirradiated at multiple locations in the nucleus with a high UV-A laser dose (40 μJ pulse^−1^) and stained for PML and gH2AX 24 h after damage induction. Interestingly, UV-induced DNA damage foci that colocalize with PML NBs are positionally more stable than non-colocalizing (14), suggesting that PML NBs may function to support topographic stability of DNA repair foci within chromatin.

Of course, these fascinating microscopic observations remain descriptive without supporting functional studies. Previously it was shown that depletion of PML indeed decreases the ability to perform homologeous recombination (HR) repair (15, 16), and it should also be mentioned that permanent lack of PML induces genomic instability and increased susceptibility to cancer (11). Thus it would be now interesting to fine-dissect the molecular mechanisms by which the presence of a PML NB is supportive to DNA repair events at particular DNA damage foci. A straightforward model would be a scaffold or platform function of the bodies for efficient biochemical repair activities nearby damaged chromatin. A combination of super-resolution techniques with live cell imaging after microirradiation is an attractive approach to further study this phenomenon.

## Optical Tweezer (OT): Erythrocyte-Mediated Force Application (EMFA)

**Figure 9:**
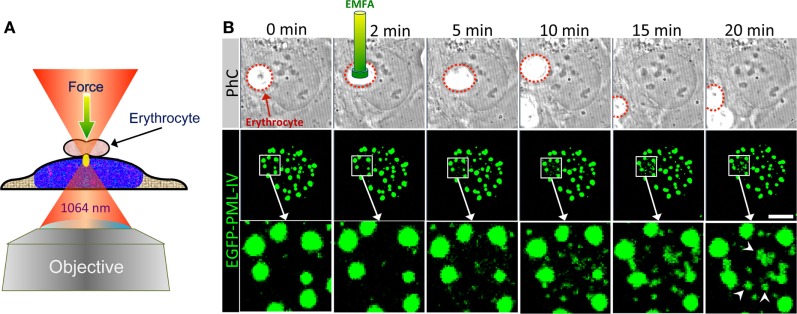
Optical tweezer (OT) as a tool to analyze PML nuclear body assembly. **(A)** Schematic depiction of erythrocyte-mediated force application (EMFA) based on OTs. Polyethylenimine coated erythrocytes attach unspecifically to the surfaces of the adherent target cells. Erythrocytes serve as very efficient “force transmitting devices” for axial force application on cells. The cell layer is moved into the region of the desired position in such a way that the laser focus (yellow ellipse) locates slightly below the erythrocyte. Immediately after switching on the laser the erythrocyte is pulled toward the focus due to the gradient force of the laser light, which causes a brief physical force onto the cell. The experimental setup used here consists of a continuous wave (cw) diode pumped Nd-YAG-laser (Spectra Physics) emitting at 1064 nm. The laser beam is coupled into an inverted confocal laser scanning microscope (LSM 510, Carl Zeiss Jena) and was focused *via* a high numerical aperture objective (100×, 1.30 NA) into the object plane. **(B)** U2OS cells expressing EGFP-tagged PML-IV were subjected to EMFA as shown in **(A)**. In phase contrast (PhC) imaging, the position of the nucleus relative to erythrocytes can be monitored during the course of the experiment (upper panels). The behavior of PML nuclear bodies was monitored by confocal sectioning (middle panels, images show maximum intensity projections). The nuclear region of force application is shown as a magnified view in the bottom panels. Arrowheads indicate *de novo* formation of PML NBs. Scale bar, 5 µm.

Since their introduction in 1986 by Ashkin et al. (153), OTs have developed rapidly over the past decades (154). OTs are today widely used tools in physics, chemistry, biological, and medical research (155). OTs are applicable to objects at nanometer up to several micrometer size ranges. The simplest form to use OTs is by focusing a laser beam using an objective lens of high numerical aperture (Figure 9A). As the rear pupil of the objective must be entirely illuminated, the diameter of the laser beam is expanded by telescope optics before directed to the microscope. Dielectric particles such as small biological objects near the focus will mainly experience two forces: radiation pressure in the direction of light propagation and gradient forces in the direction of the spatial light gradient. The balancing of both forces is required. The equilibrium position of particles in the focus is given if gradient force dominates over the scattering force.

There are also several setup variants: conventional OT with standard Gaussian laser beam, non-Gaussian laser beams based on a Bessel beam or a Laguerre–Gaussian mode, dual beams, and multiple traps or as optical strecher (156–159). OTs are excellent nanotools with which manipulation in a living cell or a living organism is possible without perforating the cell membrane. Further information on OT technologies can be found in Ref. (160–162). Generally OTs are used either to trap biological objects directly with light or as indirect force transducers to exert linear forces *via* trapped microbeads. In EMFA, polyethylenimine-coated erythrocytes are used instead of beads as the “force transmitting device” for axial force application on cells (Figure 9A) (161).

**Figure 10:**
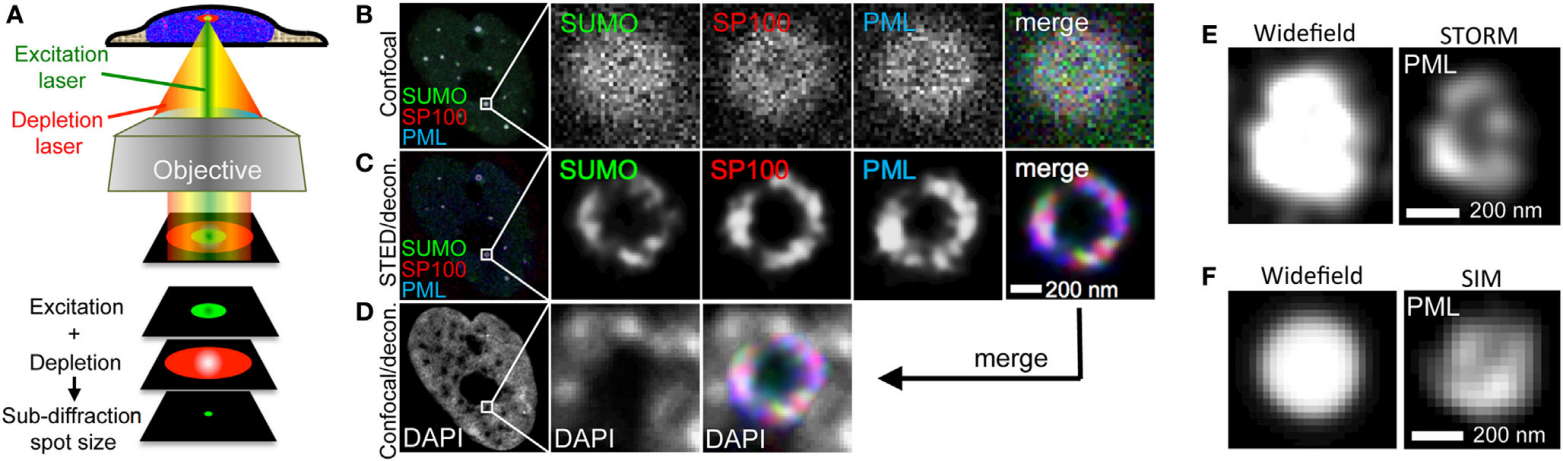
Super-resolution imaging of promyelocytic leukemia (PML) nuclear bodies. **(A)** Principle of stimulated emission depletion (STED) microscopy. In STED, two lasers are focused through a high numerical aperture objective lens. The excitation laser (green) serves to excite the fluorophore of interest similar to confocal imaging. Excitation light pulses are immediately followed by a high energy red-shifted STED beam with circularly polarized light (red). The STED light de-excites the excited fluorescence except for a small central spot due to the donut-like shape of the STED beam. This results in a subdiffraction size illumination excitation beam which can be scanned across the sample with a confocal scanner to produce super-resolved images. **(B–D)** Example of 3-color STED imaging of PML NBs using a Leica STED microscope. Fixed U2OS cells were immunofluorescently labeled to detect SUMO, SP100, and PML with secondary antibodies coupled to STAR-635P (green), STAR-580 (red), and Atto-490LS (blue), respectively. All dyes were depleted using the 770 nm STED laser. **(B)** shows one confocal section in the center of the nucleus recorded in confocal mode. **(C)** shows the same focal section as in **(B)** but recorded with the depletion laser switched on followed by deconvolution of the fluorescence signals using Huygens software (STED/decon.). **(D)** The DAPI signal was also acquired in the same focal section employing the HyVolution II mode of the Leica LSM (= confocal mode with the pinhole closed to 0.5 Airy units followed by deconvolution) (Confocal/decon.). **(E)** 3D-STORM imaging of a PML nuclear body in U2OS cells immunofluorescently labeled with an anti-PML antibody (Secondary antibody: Alexa-647N). **(F)** Super-resolution SIM imaging of a PML nuclear body in U2OS cells immunofluorescently labeled with an anti-PML antibody (Secondary antibody: Cy3). STORM and SIM imaging was performed using a Zeiss Elyra system.

Here, we used EMFA to recapitulate some of the published data on PML NB behavior after global nuclear stress. Previously it had been observed that PML NBs disintegrate into many small PML-containing structures during heat shock or exposure to Cadmium^2+^ ions, implying that these structures undergo a stress response to altered chromatin organization or topology (163, 164). When EMFA is applied on living cells expressing GFP-tagged PML, force is applied on chromatin located just below the erythrocyte (Figure 9A). This physical pressure induces the appearance of PML-containing microstructures within the region of force application (Figure 9B). Eventually, such microstructures fuse with each other to form larger structures, while the native PML NBs remain positionally stable (Figure 9B). This behavior of PML microstructures occurs on a minute scale and was interpreted as evidence for a supramolecular assembly/disassembly model in which PML NBs are not a uniform, homogeneous polymer, but rather are composed of units or modules that are linked together as supramolecular assemblies (4, 41). This view is supported by super-resolution analyses of the PML NB architecture which revealed distinct occupation rather than uniform distribution of various PML body components in a shell-like structure (35) (Figure 10). Rapid disassembly/reassembly cycles of PML nuclear bodies upon cellular stress may be instrumental in their function as damage sensors and in genome maintenance.

## Super-Resolution Microscopy (SRM)

The resolution of a light microscope is limited to about 200 nm by diffraction (165). The microscopic images of small cellular organelles or nuclear bodies in this size range therefore appear blurred and their morphological details go undetected. Fortunately, several SRM approaches have been established over past decade which improve resolution by a factor of 2–10, depending on the technique. Meanwhile, three main super-resolution technologies are commercially available, namely structured illumination microscopy (SIM), single molecule localization (SML), and stimulated emission depletion (STED) (94).

Structured illumination microscopy is a versatile and the most gentle super-resolution approach which increases the resolution by up to twofold in lateral and axial direction (166, 167). This is achieved by illuminating the sample with a grid pattern. The pattern can for example be generated by laser light passing through a movable optical grating which is projected *via* the objective onto the sample (168). The interference of the pattern with sample structures allows access to high frequency or in other words high-resolution information that would be otherwise obscured in a normal wide field image. SIM requires at least 9 images (2D-SIM) or 15 images (3D-SIM) to be taken for each optical section, whereby the illumination pattern is phase shifted and rotated in order to access the high-resolution information by sophisticated algorithms (168). The advantage of SIM is that it is compatible with all fluorescent dyes, making even super-resolved multicolor live-cell imaging feasible (169).

In SML switching of molecules between two distinct fluorescent states, normally an “on” and an “off” state is used to determine the exact position of a fluorescent molecule by determining the center of mass within the blurry fluorescent spot. The blinking is thereby adjusted to have at average only one molecule in its fluorescent state within the diffraction limited spot. The concept of blinking was realized using photoactivatable dyes, such as paGFP in photoactivated localization microscopy (PALM) and fluorescence PALM, or by using photoswitchable dye pairs (such as Cy3–Cy5 or EosFPs) as in stochastic optical reconstruction microscopy (STORM). Although PALM was established using fluorescence proteins, it was soon realized that any organic dye under appropriate reducing conditions can be brought to on and off switch cycles, a technology termed dSTORM (170).

In PALM/STORM, a series of several thousand images from the blinking specimen are recorded and mathematically processed into high-resolution images reaching resolutions below 30 nm in the lateral direction (171, 172). SML approaches have the inherent disadvantage that typically (ten)thousands of frames need to be acquired to reconstruct a single super-resolved image. The entailed low temporal resolution, extended exposure with high excitation power and associated phototoxicity render these methods less suitable for live cell imaging.

More recently developed fluctuation microscopy (SOFI, SIRF) approaches in part overcome these limitations at the expense of much lower resolution increase (173, 174). Nevertheless, live cell imaging using SML has been reported (175). Optical resolution in STED usually is well below 50 nm in fixed samples and ca. 70 nm in live-cell experiments (94). A more in depth explanation on the theory and on practical applications of SIM, SML, and STED can be found here: (176, 177). Possible practical limitations and compromises that must be considered when designing super-resolution experiments have been pointed out by Lambert and Waters (178).

Stimulated emission depletion is based on the application of two laser beams in a confocal (point-scanning) set-up. The STED depletion laser is delivered into the optical path through a phase filter, which creates a donut-shaped beam on the confocal fluorescence spot by controlled de-excitation of the previously excited fluorophore (Figure 10A). The high intensity STED beam extinguishes the peripheral fluorescence signal, leaving a subdiffraction-sized fluorescence spot in the center of the donut (179, 180) (Figure 10A). Interestingly, the first report on super-resolution light microscopy of PML nuclear bodies was not based on the three SRM methods described above but was realized with the so-called 4Pi microscope developed by Hell et al. (179). Four-Pi fluorescence laser-scanning microscopy studies revealed that during interphase PML NBs adopt a spherical organization characterized by the assembly of different PML body components into distinct, partially overlapping patches within a 50–100-nm thick shell (35). The spherical organization of PML NBs had been observed already before by electron microscopy (36, 48), but 4Pi allowed for simultaneous pair-wise detection of two PML body components.

One example of STED nanoscopy on PML NBs is shown in Figures 10B–D. The three major PML NB constituents SUMO, SP100 and PML were immunolabeled in U2OS cells with different fluorophores and imaged in confocal as well as in STED mode to visualize the improvement in optical resolution through STED (Figures 10B,C, respectively). As expected, STED reveals that these proteins decorate distinct, yet partially overlapping patches in the peripheral shell of the PML NB (Figure 10C). Super-imposition of the STED image with the DAPI pattern of the same confocal section confirms the absence of chromatin in the core of normal PML NBs (Figure 10D) (36). We also applied STORM and SIM imaging of PML in U2OS cells. STORM is similarly well suited to reveal the shell morphology of PML protein distribution (Figure 10E) while the resolution in SIM is, as expected, considerably lower than in STED or STORM (Figure 10F). However, since the laser load is much less, SIM would be better suited for live cell super-resolved imaging of PML nuclear body morphology, i.e., in the analyses of fission and fusion events of PML microstructures in stress conditions (Figure 9) or DNA at damage foci (Figure 8). In conclusion, this section shows that with commercially available SRM microscopes the analysis of biomolecules can be lifted to a new optical dimension.

## Outlook

We believe that many biochemical or molecular biology oriented research labs are still not aware of the multitude of new and exciting microscopic methods and their capabilities. The aim of this contribution was to present recent advances in bioimaging in combination with selected application examples in PML nuclear body biology. Here we have illustrated the power of imaging methods and provide a guide to these techniques to make them more accessible to a larger number of labs involved in oncogene or tumor suppressor research. We have presented several experimental examples feasible in our imaging facility, yet the number of additional techniques is much higher. Bioimaging facility networks have been established in several countries worldwide and these can be approached with specific imaging requests. A source for comprehensive bioimaging methodology is available Europe-wide^4^ and a global bioimaging network project may be realized in the near future.^5^

Local, regional, national, and supranational imaging networks will continue to develop with the aim to provide access, service and training to state-of-the-art imaging technologies. Only such dedicated facility infrastructures and/or very specialized imaging research labs will be able to cope with the fast development of novel microscopy techniques. Although probably a demanding task, both, the facility members as well as basic research scientists are now in charge to synergistically work together to fully exploit the powerful imaging tools in the study of molecular and cellular mechanisms.

We have also summarized the current knowledge on the potential functions and assembly of PML nuclear bodies. PML has been analyzed using wet-lab and genetic techniques on one hand and imaging methods on the other. With the new microscopy methods now at hand it will be exciting to see the two different approaches merging. For example, a combination of STED and FCS (STED-FCS) (181), should make it possible to assess biophysical and binding properties of PML-interacting partners within PML NBs. This would help (i) to understand the molecular/biochemical events occurring molecularly at PML NBs at sites of DNA damage and (ii) to better visualize/understand the proposed phase separation function of PML NBs (68). For example, single-molecule tracking at nanoscale resolution has recently been employed to demonstrate the liquid droplet nature of stress granules in the cell nucleus (182). As nanoscopy will become less phototoxic in the future (183), super-resolution imaging of APBs in living cells will shed more light on the mechanisms of DNA recombination events which occur in PML NBs during telomere elongation in ALT cancer cells (184).

More physiologically, it would be seminal to investigate PML NBs in their most physiological setting, the living model organism. A combination of confocal microscopy and/or nanoscopy with adaptive optics for better tissue penetration (185, 186) would enable monitoring of fluorescent PML NBs in living tissue such as skin or brain of GFP-PML knock-in mice under normal vs. stress conditions (irradiation, chemicals). Since PML has an established role in certain stem cell niches, these nuclear bodies could be imaged and functionally analyzed in various living spheroid or organoid stem cell systems using a combination of multicolor lightsheet and super-resolution approaches (187, 188). In the same experimental setting, laser-assisted ablation of single PML NB-expressing cells could help to identify PML-mediated mechanisms of stem cell plasticity (189). Seeing is believing and therefore we look forward to monitor PML nuclear body biochemistry through new imaging set-ups in real time in living cells in the future.

## Author Note

This contribution is dedicated to Jörg Langowski.

## Author Contributions

CH, SM, KW, and PH prepared the figures and wrote the text.

## Conflict of Interest Statement

KW was employed by Carl Zeiss Microscopy GmbH (ZEISS Group, Carl-Zeiss-Promenade 1007745 Jena, Germany). All other authors declare no competing interests.

## Footnotes

^1^http://npd.hgu.mrc.ac.uk/ (Accessed: November 17, 2017).

^2^https://thebiogrid.org/111384/summary/homo-sapiens/pml.html (Accessed: November 17, 2017).

^3^http://www.lfd.uci.edu/globals/ (Accessed: November 17, 2017).

^4^http://www.eurobioimaging.eu/ (Accessed: January 12, 2018).

^5^http://www.eurobioimaging.eu/global-bioimaging (Accessed: January 12, 2018).
